# Vapor-Assisted Conversion of Heterobimetallic Titanium–Organic Framework Thin Films

**DOI:** 10.1021/acs.chemmater.3c01389

**Published:** 2023-12-11

**Authors:** María Romero-Ángel, Víctor Rubio-Giménez, Eloy P. Gómez-Oliveira, Margot F. K. Verstreken, Jorid Smets, Jesús Gándara-Loe, Natalia M Padial, Rob Ameloot, Sergio Tatay, Carlos Martí-Gastaldo

**Affiliations:** † Instituto de Ciencia Molecular (ICMol), Universitat de València, Catedrático José Beltrán 2, 46980 Paterna, Spain; ‡ Centre for Membrane Separations, Adsorption, Catalysis and Spectroscopy (cMACS), Katholieke Universiteit Leuven, Celestijnenlaan 200F, 3001 Leuven, Belgium

## Abstract

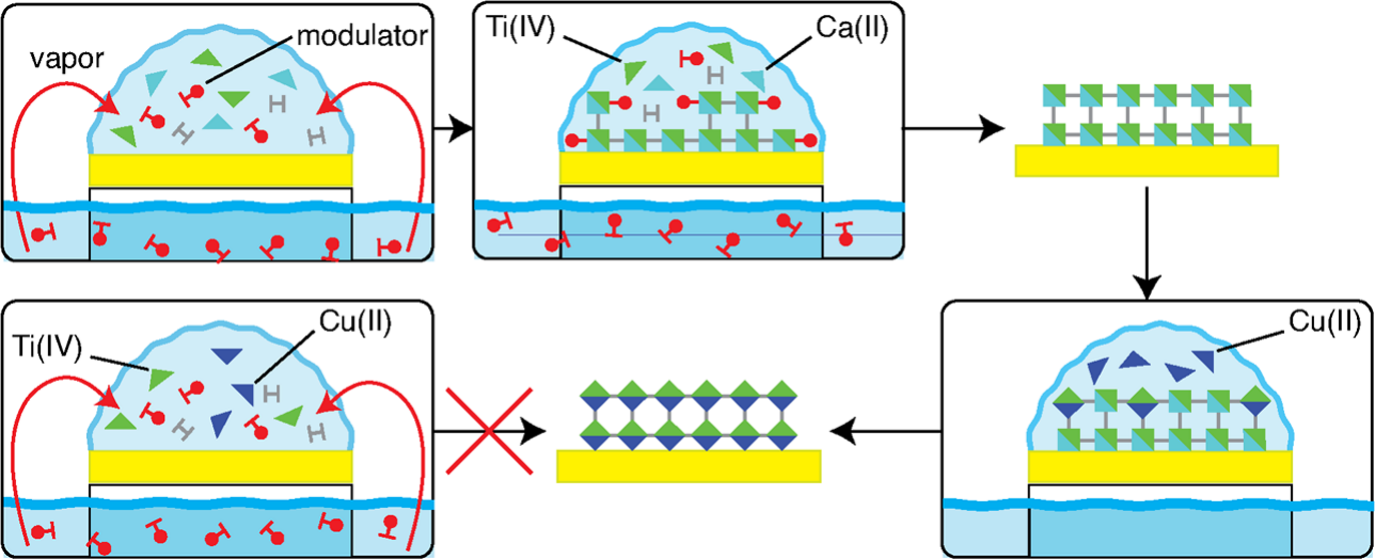

Heterobimetallic Metal–Organic Frameworks (MOFs) synergically combine the properties of two metal ions, thus offering significant advantages over homometallic MOFs in gas storage, separation, and catalysis, among other applications. However, these remain centered on bulk materials, while applications that require functional coatings on solid supports are not developed. We explore for the first time the deposition of heterometallic Ti-based MOF thin films using vapor-assisted conversion on substrates functionalized with a self-assembled monolayer. Furthermore, metal-induced dynamic topological transformation allows the conversion of MUV-10(Ca) films into MUV-101(Co) and MUV-102(Cu), which is not accessible through direct synthesis, without morphologically altering the films. These nonconventional thin-film deposition techniques enable homogeneous and crystalline coatings of heterometallic titanium MOFs that also maintain their corresponding porosity.

Metal–Organic Frameworks (MOFs) are crystalline porous architectures composed of organic linkers and inorganic metal nodes/clusters. These molecular frameworks combine structural and compositional diversities beyond comparison with other synthetic materials with high intrinsic porosities.^1^ Because of these characteristics, MOFs are very convenient materials to use in a wide range of different applications.^2,3^

The processing of MOFs as thin films offers increasing advantages for some of those applications, such as energy conversion,^4^ electronic devices,^5^ sensor technology,^6^ or catalysis.^7^ Driven by such potential, the fabrication of porous thin films has seen great progress, and several surface deposition techniques have been developed or applied to MOFs.^8−11^ For instance, MOF thin films have been prepared from preformed materials using drop-casting, dip-coating, spin-coating, inkjet printing, or electrophoretic deposition.^12−16^ Alternatively, films have been assembled directly on the surface from metal-linker solutions via solvothermal synthesis or via an electrochemical reaction, either using the anode as the metal source or the cathode to reduce and deprotonate the linker.^17,18^ Otherwise, MOF coatings can be crystallized on a surface from precursor droplets via electrospraying or aerosol jet printing.^19,20^ Film growth can also be achieved by confining the reaction to the gas–solid interface through chemical vapor deposition, thus avoiding the use of solvents.^21^ Vapor-assisted conversion (VAC) is another thin-film fabrication methodology at the edge of vapor and solution synthesis.^22−26^ Alternatively, the assembling of the MOFs can be carried out in several steps, the so-called layer-by-layer (LbL) or liquid phase epitaxy (LPE) approach, which involves the sequential growth of the ultrathin film by consecutive exposure of the substrate to the different building blocks.^27,28^ Beyond the chosen film-making method, there are several factors that influence film growth such as the surface pretreatment, the choice of the modulator, the concentration of the precursors, the time, or the reaction temperature.^29,30^

The complexity of these frameworks has recently increased via the controlled combination of different organic and inorganic building blocks. The organic approach to multivariance involves the combination of linkers featuring varying functional groups but with fixed length and connectivity, often having a small impact on the construction of multivariate (MVT) MOFs.^31^ In turn, the combination of different metals into mixed-metal or heterometallic MOFs is challenging due to differences in polarizing power, acidity, or coordination geometries for the various metals.^32−34^ This is likely why most MOF films in the literature are based on homometallic frameworks, while heterometallic ones are few in comparison, typically deposited electrochemically or via hydrothermal synthesis.^35−38^ This limitation is even more critical for titanium(IV) metal ions, whose intrinsic photoactivity is ideal for designing photocatalytic MOFs as demonstrated by Serre, Sanchez, Férey, and co-workers with the archetypical MIL-125.^39^ Its strong polarizing power and challenging chemistry in solution limits the formation of porous, crystalline architectures even for single metal nodes.^40^ Nevertheless, the synthesis of heterometallic titanium MOFs has been frequently achieved, either via direct solvothermal synthesis in a one-pot reaction or by indirect postsynthetic metal exchange.^34^ Over the last years, we have reported the families of titanium–organic frameworks: MUV-10,^41^ MUV-101, and MUV-102^42^ (MUV = Materials Universitat de València), all based on benzene-1,3,5-tricarboxylate (btc) linkers with formulas [Ti_2_M_2_(μ_3_-O)_2_(btc)_2.66_(H_2_O)_4_] (M = Ca, Mn, Ba, Sr, Cd),^43^ [TiM_2_(μ_3_-O)(btc)_2_X_3_] (M = Mg, Fe, Co, Ni; X = H_2_O, OH-, O^2–^), and [Ti_0.4_M_1.6_(O)_0.4_(btc)_2_(H_2_O)_1.6_] (M = Cu). These families can also be built by direct solvothermal synthesis and indirect metal-exchange routes. Their Ti_2_M_2_, TiM_2_, and TiM heterobimetallic clusters display an ordered combination of Ti^4+^ and first-row transition metals either localized by crystallographically distinct coordination environments [i.e., MUV-10(Ca)] or statistically distributed at the cluster level [i.e., MUV-101(Co) and MUV-102(Cu), Figure 1]. This controlled cluster composition allows features not accessible to homometallic MOFs, such as synergistic dual-metal catalysis,^44,45^ tailorable electronic structure for engineering of photocatalytic performance,^41,43^ controlled grafting of small molecules,^46^ and solid-state reactivity,^42,47^ all enabled by the additional metal.

**Figure 1:**
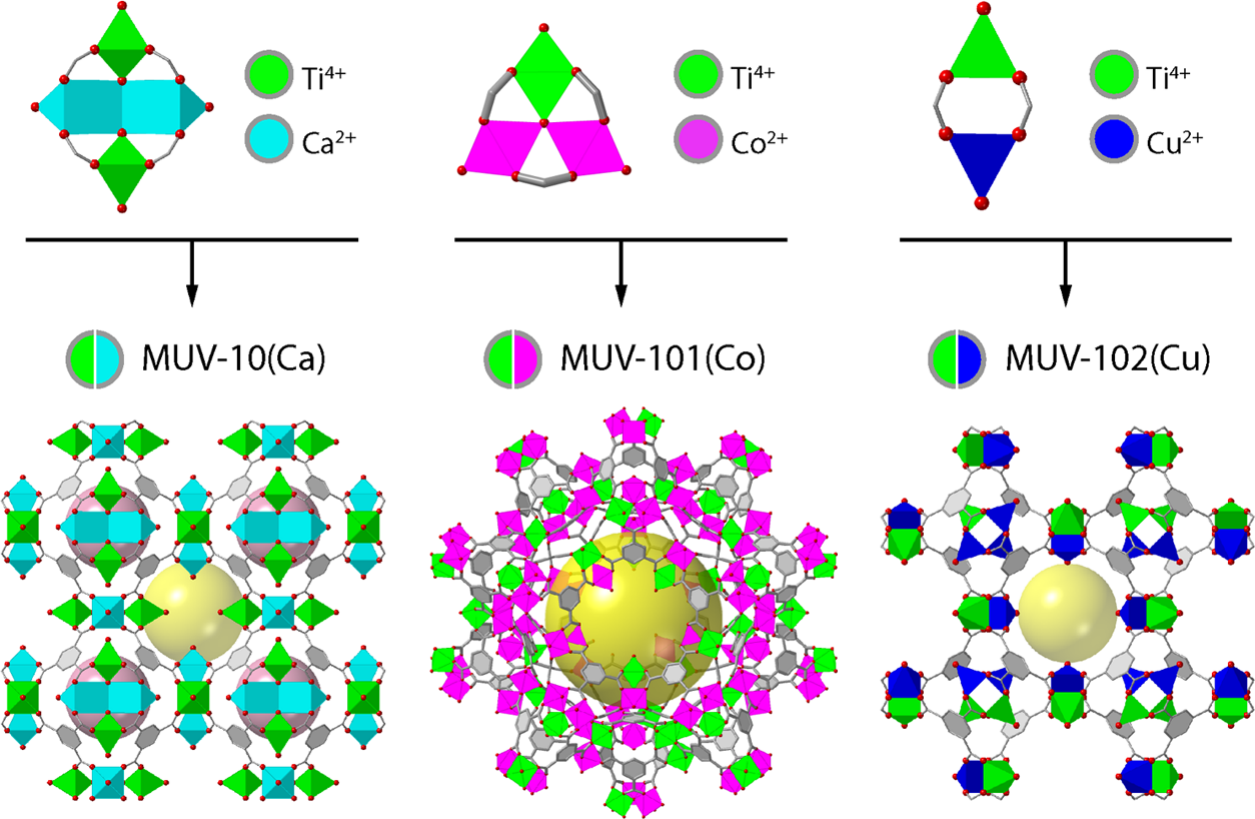
Heterobimetallic titanium MOFs used here for thin-film fabrication. From left to right, secondary building unit, chemical composition, and structure of MUV-10(Ca), MUV-101(Co), and MUV-102(Cu) frameworks.

Provided successful thin film fabrication, these frameworks would be particularly appealing for energy conversion applications, such as electrocatalysis, photocatalysis, or photoelectrocatalysis, based on their excellent chemical stability and the synergistic interaction of Ti^4+^ with other metals. In this work, we introduce a hybrid approach that combines VAC and metal-exchange reactions to produce crystalline and porous heterometallic thin films of MUV-101(Co) and MUV-102(Cu), the latter not accessible through direct synthesis methods, by using a MUV-10(Ca) film as prepatterned precursor.

First, we attempted the fabrication of heterometallic MUV-101(Co) films through conventional solvothermal thin-film deposition by adapting the previously reported synthesis procedure.^44^ A gold-coated silicon substrate was placed in a 25 mL Schott bottle and reacted with titanium(IV) isopropoxide, btc, and the corresponding chloride metal salt in a mixture of N,N-dimethyl-formamide (DMF) and acetic acid (AcOH) at 120 °C during 48 h. These harsh synthetic conditions lead to the partial delamination of the gold layer despite the Cr adhesion layer. In search of a viable strategy to produce homogeneous thin films of a heterometallic MOF, we finally focused on the VAC method first introduced by Medina and co-workers.^22^

As schematized in Figure 2a, substrates coated with a drop of the precursor’s solution are exposed to modulator/solvent vapor mixtures. By controlling the concentration of the vapor precursors, reaction/nucleation rates can be modified to obtain homogeneous high-quality films. VAC proved to be particularly well suited for the growth of heterometallic MUV-101(Co) films due to the possibility of introducing the required acid modulator in the vapor phase. This conveniently avoids exposure to significantly higher concentrations of the acid in solution, which damages the substrate. The reported VAC procedure for MUV-101(Co) was adapted by placing a gold-coated substrate above a mixture of DMF (2.5 mL) and AcOH (0.5 mL) inside a 25 mL Schott bottle with the help of Raschig rings. Next, the substrate was covered with a 50 μL drop containing the metal ion precursors (3 μL of Ti(Oi Pr)_4_ and 2.6 mg of CoCl_2_) and the ligand (10.4 mg of btc), previously dissolved in 0.6 mL of AcOH and 1 mL of DMF. Finally, the bottle was sealed and heated at 100 °C for 6 h. Afterward, the substrate was removed and washed with DMF and methanol, followed by drying under an N_2_ stream. As visible in Figure 3a–d, optical and scanning electron microscopy (SEM) images showed 2.8 μm thick films of 0.4 μm octahedral particles with good coverage and reasonable homogeneity over micrometric areas. Energy dispersive X-ray (EDX) analysis of the films produced the expected 1:2 Ti/Co metal ratio. However, the crystallinity of the resulting film was fairly poor; even synchrotron grazing incidence X-ray diffraction (GIXRD) showed very low intensity MUV-101(Co) peaks (Figure S1). Compared to the crystallinity of the thin films of UiO-type MOFs prepared by this method,^48^ we argued the assembly of TiM_2_ clusters might be imposing additional limitations to crystallization.

**Figure 2:**
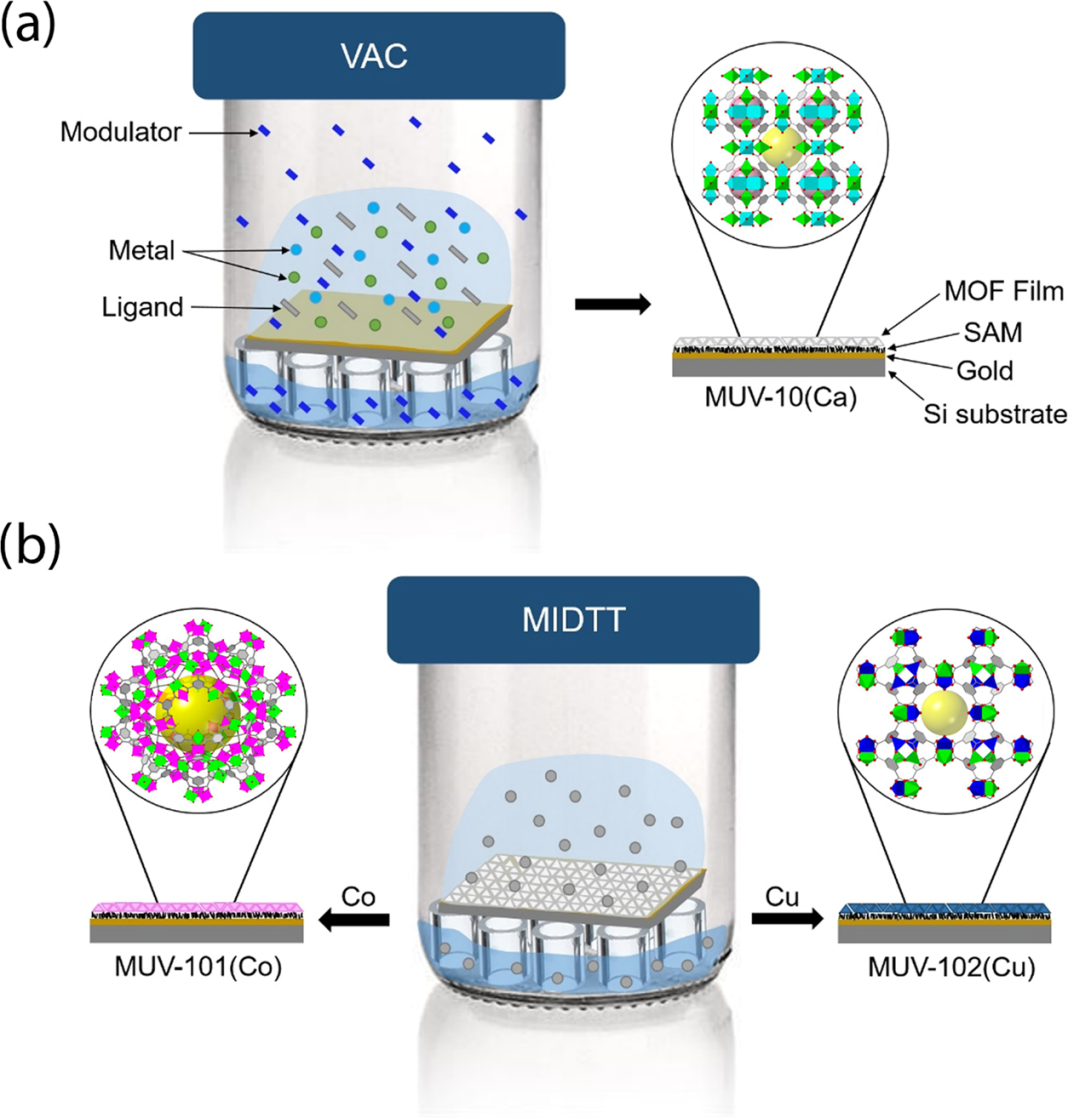
Schematic representation of (a) VAC method for MUV-10(Ca) thin film fabrication and (b) metal-induced dynamic topological transformation (MIDTT) process for the transformation of a MUV-10(Ca) film into MUV-101(Co) and MUV-102(Cu) films.

**Figure 3:**
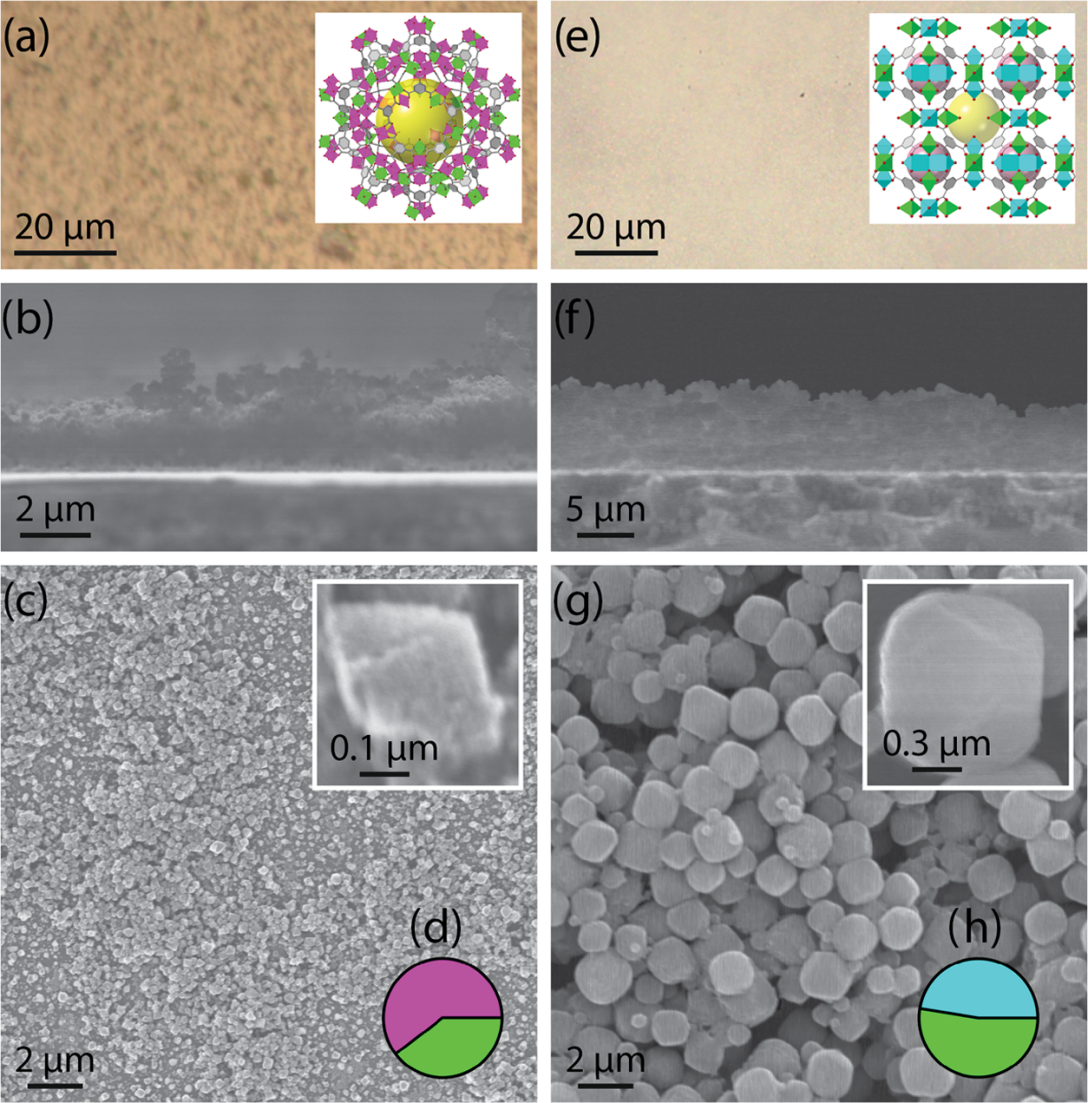
MUV-101(Co) (left) and MUV-10(Ca) (right) films prepared using the VAC method. (a, e) optical images and bulk crystal structure; (b, f) cross-sectional SEM images; (c, g) top SEM images; and (d, h) EDX metal composition (color code: Co, pink; Ca, blue; Ti, green).

This encouraged us to explore other alternatives that were not reliant on the assembly of the framework from its molecular precursors and involved the transformation of other frameworks acting as seeding MOF. We recently reported how several heterometallic MOFs, such as the MUV-101 family (Fe, Co, Ni, Zn) and MUV-102(Cu), can all be prepared starting from MUV-10(Ca) in a process dubbed as MIDTT.^42^ This simple method consists of incubating MUV-10(Ca) in a methanol solution containing the desired first-row transition metal at 65 °C. Interestingly, MIDTT enabled the production of MUV-101(Zn) and MUV-102(Cu), which are not accessible through direct solvothermal synthesis. Framework transformation proceeds at the single particle level and is controlled by the metal in solution. This dictates the evolution of the 8-connected (8-c) Ti_2_M_2_ clusters in the ***the*** net of MUV-10 into 6-c TiM_2_ trimers or 4-c TiM paddlewheel dimers for the formation of ***mtn*** MUV-101 or ***tbo*** MUV-102 nets (Figure 1). MUV-10(Ca) can be easily synthesized in high yields from the direct reaction of CaCl_2_ with several titanium precursors in a relatively wide range of experimental conditions.^49^ At this point, MIDTT was chosen as a softer and more amenable method for the preparation of heterometallic MOF thin films, both as an alternative to the high-temperature VAC method for MUV-101(Co) and to access frameworks that are not obtainable via direct synthesis. Thus, we set out to prepare MUV-10(Ca) thin films through VAC over gold-coated substrates as a first step before the MIDTT. Unfortunately, adapting the same VAC conditions of MUV-101(Co) (SI Section S4.1.1) led to inhomogeneous MUV-10(Ca) films with incomplete coverage (see Figure S2). Attempts to grow the films using the same conditions and different substrates (bare silicon wafers instead of gold-coated ones) led to similar results (Figures S3 and S4). The presence of cracks in the final films could be the result of evaporation-induced strain during film drying.^50^ In an attempt to minimize that effect, we prefunctionalized the gold-covered substrates with different self-assembled monolayers (SAMs): 1-octadecanethiol, 4-mercaptopyridine, and 16-mercaptohexadecanoic acid (SI Section S4.1.2). As can be seen in Figures S5 and S7, only pyridine-functionalized substrates yielded crystalline and continuous MUV-10(Ca) thin films for which EDX analysis showed a Ca/Ti ratio close to the theoretical one. However, SEM images (Figure S5a) showed somewhat inhomogeneous films formed by ill-defined particles of about 300 nm. Thus, we explored different reaction temperatures and times, precursor concentrations, and modulators (acetic, formic, or benzoic acid, Table S1) in order to maximize the film quality in terms of crystallinity, homogeneity, and metal-to-metal ratio. The variation of the drop volumes proved to be an important factor. If a large droplet (≥100 μL) is laid on top of the substrate, the film tends to form on the edges of the substrate, leaving a hole in the center. Regarding precursor concentrations, no material was formed when the millimolar range was exceeded. From all explored modulators (benzoic, formic, and acetic acids, SI Section S4.1.4), only AcOH produced a high-quality crystalline film. But when changing the temperature and reaction time (SI Section S4.1.5), formic acid can also yield a crystalline and homogeneous MUV-10(Ca) film with a Ca/Ti ratio close to the theoretical one (Figure S14). This demonstrates the versatility of the MUV-10 synthesis, which works under different conditions. Only minor changes are required to adapt the bulk synthesis to films. The highest film quality as determined by specular XRD was obtained using 4-mercaptopyridine functionalized gold-coated substrates, AcOH as a modulator, and 6 h of reaction time at 100 °C (Table S1, **entry 6**, SI Section S4.2.1). The high crystallinity of the film, which matches the simulated pattern of MUV-10(Ca), was verified by specular XRD (Figures S15b and S16). Remarkably, these conditions are milder compared to those of the bulk material.^49^ Furthermore, As shown in Figures 3e–h and S17, optical and SEM imaging showed continuously covered substrates with a MUV-10(Ca) layer of ca. 8 μm formed by aggregated crystallites. These truncated octahedral particles of 1.5 μm in size are smaller than the 10 μm octahedral microcrystals first described for bulk MUV-10.^49^ This is not surprising, as it is well known that crystal shape and size are heavily dependent on the reaction conditions and can also be influenced by the substrate.^10,51^ EDX analysis also confirmed a composition close to the ideal 1:1 Ti/Ca ratio (Figure 3h).

Next, following the previously described MIDTT procedure as schematized in Figure 2b,^42^ we exposed a series of MUV-10(Ca) films to a solution of Co(NO_3_)_2_ in methanol at 65 °C and followed the transmetalation process to MUV-101(Co) by using EDX and synchrotron GIXRD. EDX (Figure 4a, Table S2) shows metal ratios evolved within days from those of MUV-10 (Ti:Ca 1:1) to those expected for MUV-101(Co) (Ti:Co 1:2), thus indicating the fulfillment of the MIDTT process. In fact, we found that the transmetalation follows first-order rate kinetics (Figure 4a). Considering that in the MIDTT procedure, metal solutions are neither washed nor replenished, we calculated the corresponding reaction rate constants (Table S3). Synchrotron GIXRD (Figure 4b) further demonstrates that after 15 days, the structural MIDTT transformation is completed. From that time onward, diffractograms only feature the characteristic MUV-101(Co) peaks, with no MUV-10(Ca) peaks remaining. During the transmetalation process, the film color also evolved from white to the characteristic light pink of MUV-101(Co) while maintaining the film’s homogeneous coverage and thickness (Figures 4c–eand S19). On the particle, the shape evolved from truncated to regular octahedra, and the average particle size was reduced to 900 nm. Furthermore, in order to demonstrate the uniform distribution of elements throughout the entire film, we examined the cross-sectional SEM-EDX elemental mapping. As visible in Figure S20, MIDTT converted MUV-101(Co) films show a homogeneous distribution of cobalt and titanium through the entire thickness and across a large micrometric area, without significant presence of remaining calcium. The element distribution at the single crystallite level was also closely examined by focused ion beam field emission scanning electron microscopy (FIB-FESEM) and EDX mapping. Here, larger crystals from a bulk MUV-10(Ca) sample were cut and analyzed at initial (0 days), intermediate, and completed MIDTT stages to MUV-101(Co) (Figure S21). As we previously reported, the transformation occurs at the single crystallite level without redissolution. At a very early stage, the slow diffusion of the incoming metal ions in solution provokes a slight concentration gradient from the external surface to the inside of the crystals, although this is only visible at a higher resolution and under slower transformation regimes.^42^ Nevertheless, this gradient dissipates as the MIDTT advances, and it is not clearly visible anymore at the intermediate stage (Figure S21), which shows an even distribution of metals along the entire crystal. The same can be observed at the final stage of the transformation when a calcium signal is no longer observed in the EDX mapping (Figure S21).

**Figure 4:**
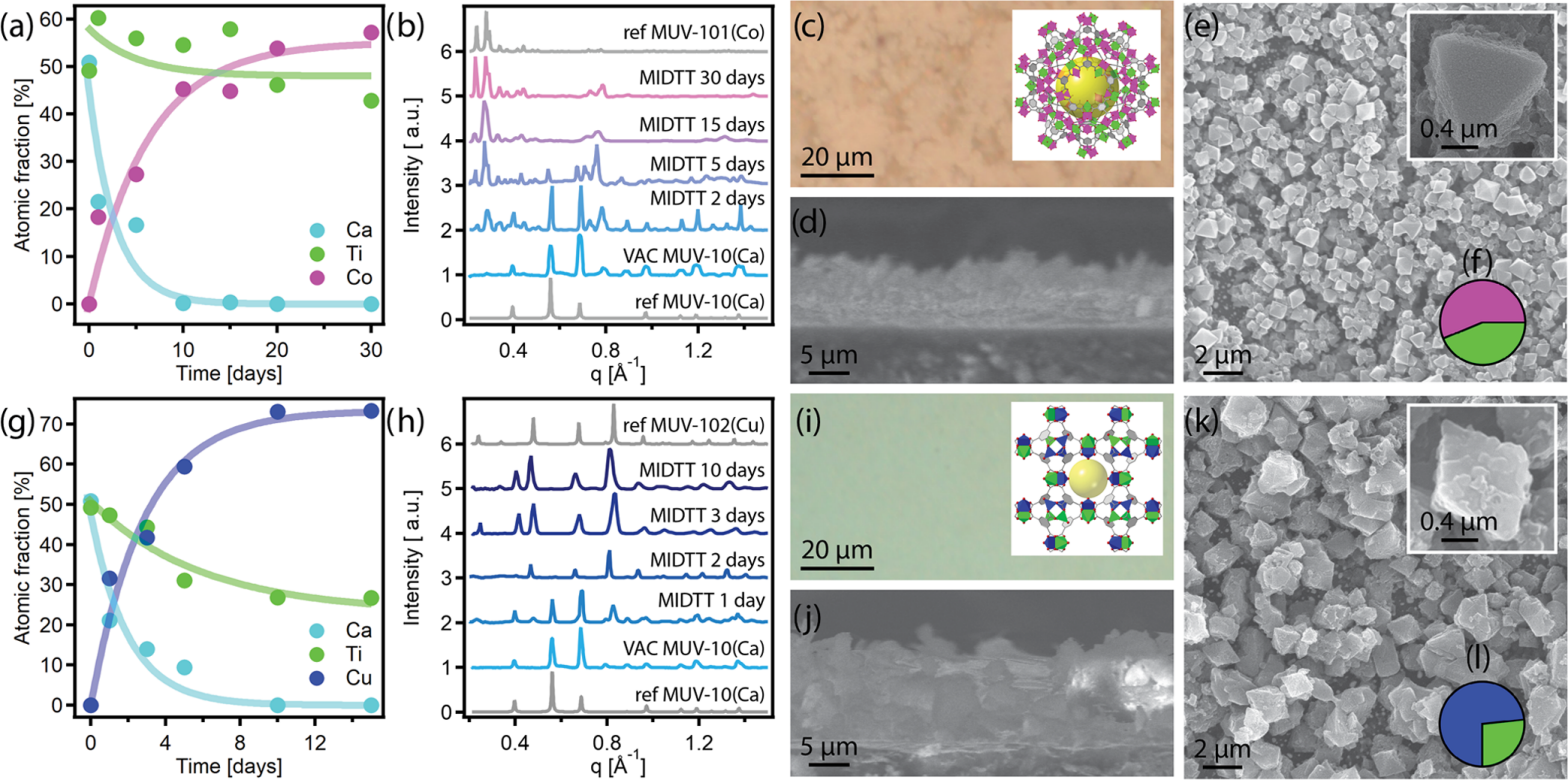
MUV-101(Co) (top) and MUV-102(Cu) (bottom) films fully transformed from MUV-10(Ca) using the MIDTT method. (a, g) EDX metal composition as a function of transmetalation time. Data was fitted to first-order rate kinetics for each individual metal atomic fraction percentage according to equations [AF%] = [AF%]_0_·*e*^–*kt*^ for Ca and Ti; and [AF%] = [AF%]_0_ – [AF%]_0_·*e*^–*kt*^ for Co and Cu; (b, h) GIXRD evolution as a function of time, diffractograms have been baselined for easier comparison; (c, i) optical images and bulk crystal structure; (d, j) lateral SEM images; (e, k) SEM images; and (f, l) EDX metal composition of the fully transformed films (pink: Co; dark blue: Cu; green: Ti).

Besides MUV-10(Ca) transformation into MUV-101(Co), we also explored MIDTT into MUV-102(Cu). Contrary to MUV-101(Co), MUV-102(Cu) is not accessible via direct synthesis, leaving MIDTT from a VAC MUV-10(Ca) precursor film as the only route for MUV-102(Cu) thin films. Results of exposing MUV-10(Ca) films to methanolic Cu(NO_3_)_2_ solutions in the same conditions as those used for MUV-101(Co) are summarized in Figure 4g–k. As in the case of bulk MIDTT, transformation of MUV-10 into MUV-102(Cu) was faster than that of MUV-101(Co) and took only 2–3 days, as indicated by EDX data (Figure 4g, Table S4), which also follows first-order rate kinetics with rate constants of generally larger values than the previous case (Table S5). Synchrotron GIXRD (Figure 4h) also matches the EDX timeline, with patterns exclusively matching those of bulk HKUST-1 after 3 days. An equivalent transition of the coating from white to a bluish color occurred. SEM and optical microscopy images (Figures 4i–kand S22) show that the film homogeneity, coverage, and thickness are generally maintained from MUV-10(Ca). Films are formed by the aggregation of 1.8 μm octahedral particles, slightly larger in size than in the case of MUV-101(Co). Cross-sectional SEM-EDX mapping (Figure S23) also shows a homogeneous distribution of copper and titanium without a significant presence of remaining calcium throughout the entire thickness.

Finally, we confirmed the porous nature of the resulting films before and after the complete MIDTT transformations using Kr physisorption.^52^Figure 5 features the Kr adsorption/desorption isotherms measured at 77 K for the MUV-10(Ca), MUV-101(Co), and MUV-102(Cu) samples after activation at 120 °C for 12 h. The three MOF films feature type I isotherms according to the IUPAC classification,^53^ thus corroborating the microporous nature of the MOF layers and no significant presence of mesoporosity from interparticle gaps. The increasing Kr uptakes and film surface areas from MUV-10(Ca) to MUV-101(Co) or MUV-102(Cu) are consistent with the MIDTT transformations in similar scales to that observed in the bulk phase.^42^ Thus,we can confirm the effective transfer of the accessible porosity from the bulk material to the film for both MOFs.

**Figure 5:**
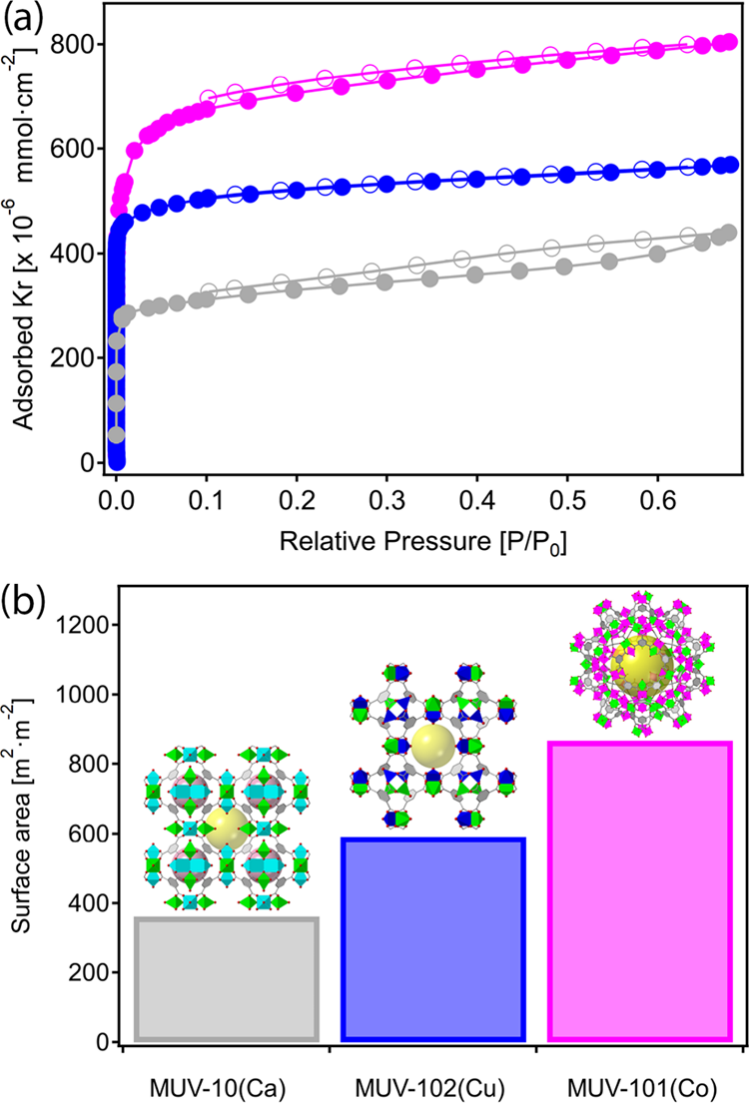
Kr physisorption of the VAC MUV-10(Ca) film, together with MUV-101(Co) and MUV-102(Cu) films fully transformed from MUV-10(Ca) using the MIDTT method. (a) Kr adsorption (full circles)/desorption (empty circles) isotherms measured at 77 K. (b) Film surface areas as calculated from the multipoint BET surface areas and the total geometrical areas of the film samples.

In conclusion, we addressed the fabrication of high-quality thin films of titanium-based heterometallic MOFs for the first time by using various growth methods. Our results indicate that VAC is compatible with the deposition of heterometallic MOFs, producing more homogeneous and crystalline thin films than conventional techniques. The combination of VAC in SAM-functionalized substrates with MIDTT introduces an alternative approach toward heterometallic MOF thin-film formation that makes use of metal-induced topological transformations, hence allowing access to MOFs not reachable via direct synthesis. Based on our preliminary results on the design of additional reticular frameworks with the MIDDT approach and the exciting opportunities offered by heterometallic titanium clusters for advanced functionalities, this work opens the door for the exploitation of this family of materials in the fabrication of electrodes and photoelectrodes of interest in energy conversion applications.

## Experimental Section

### Gold-Coated Silicon Substrate Preparation

Prior to evaporation of Au, Si substrates were soaked in a fresh solution of acetone and sonicated for 5 min, followed by isopropanol for an additional 5 min. A 3 nm Cr layer as adhesive, followed by a 10 nm Au layer, was then evaporated in an Edwards Auto 500 thermal evaporator using a tungsten basket coated with Al_2_O_3_ placed inside a nitrogen glovebox. The base pressure was 1.5 × 10^–6^ mbar, and the evaporation rate was 0.02 nm s^–1^.

### Functionalization of SAMs

10 min of O_2_ plasma cleaning (MiniPCFlecto, Plasma Technology) was conducted prior to the immersion in a 1 mM ethanol solution of 4-mercaptopyridine at room temperature for 12 h. The SAM-functionalized substrates were washed with fresh ethanol and afterward stored in ethanol until their utilization in MOF film synthesis. Immediately before use, the SAM-functionalized substrates were rinsed with fresh ethanol and dried under a stream of nitrogen. For the functionalization other SAMs, substrates were previously activated via O_2_ plasma treatment for 10 min and then immersed in a 1 mM ethanol solution of 1-octadecanethiol or 16-mercaptohexadecanoic acid for 16 h. In the case of the 16-mercaptohexadecanoic acid, the ethanol solution contains 10% AcOH to avoid the formation of hydrogen-bond bilayers. Again, immediately before use, the SAM-functionalized substrates were rinsed with fresh ethanol and dried under a stream of nitrogen.

### MUV-101(Co) Solvothermal Films

A gold-coated substrate was placed on the bottom of a 25 mL glass bottle (Schott Duran, borosilicate 3.3, ISO4796, 100 mL) with a PBT cap equipped with a Teflon seal. The bottle was filled with the precursors of the published material MUV-101(Co).^1^ In a typical experiment, 595 μmol of btc (125.0 mg) and 240 μmol of MCl_2_ (M = Mg, Fe, Co, or Ni) were dissolved in a mixture of 12 mL of DMF and 7 mL of AcOH. Next, 36 μL of Ti(Oi Pr)4 (120 μmol) was added to the solution in a glovebox. The bottle was put in an oven at 120 °C for 48 h (heating rate: 2.0 °C·min^–1^; cooling rate: 0.4 °C·min^–1^). Afterward, the substrate was removed from the synthesis mixture, washed with DMF and methanol, and finally dried under a nitrogen stream.

### MUV-101(Co) VAC Films

In a typical experiment, a glass bottle (Schott Duran, borosilicate 3.3, ISO4796, 100 mL) with a PBT cap equipped with a Teflon seal was used. The bottom part of the bottle was filled with 14 Raschig rings (10 mm × 10 mm, soda–lime glass) to obtain an elevated flat platform for the substrate. A mixture of DMF (2.5 mL) and AcOH (0.5 mL) was added. Afterward, a substrate (1 cm × 1 cm) was placed on top of the Raschig rings and fully coated with a drop (50 μL) of a freshly prepared MOF precursor solution. The bottle was closed and then transferred into an oven preheated at 100 °C, where it was kept for 6 h. Afterward, the bottle was removed from the oven and allowed to cool for 10 min before the substrate was taken out, washed with DMF and methanol, and finally dried under a nitrogen stream. For the precursor solution, 3 μL (10 μmol) of Ti(Oi Pr)_4_, 2.6 mg (20 μmol) of CoCl_2_·6H_2_O, and 10.4 mg (50 μmol) of btc were dissolved in 1 mL DMF and 0.6 mL of AcOH by ultrasonic treatment.

### MUV-10(Ca) VAC Films

The same procedure as that for MUV-101(Co) VAC films was used. But in this case, for the precursor solution, 9 μL (3 mmol) of Ti(Oi Pr)_4_, 5 mg (3 mmol) of CaCl_2_·6H_2_O, and 32.4 mg (14.875 mmol) of H_3_btc were dissolved in 1 mL of DMF and 1.8 mL (1000 equiv) of AcOH by ultrasonic treatment.

### MUV-101(Co) and MUV-102(Cu) Films by MIDTT

Similarly to the VAC procedure described above, the bottom part of the bottle was filled with methanol. Next, the MUV-10(Ca) film was placed on top of the Raschig Rings and fully covered with a drop of freshly prepared solution of the corresponding metal (0.2 M of M(NO_3_)_2_·*x*H_2_O in methanol, M = Co, Cu). The bottle was then closed and transferred into an oven preheated at 65 °C for different time periods (see S4 and S5 sections). Afterward, the bottle was removed from the oven and allowed to cool down for 10 min before the substrate was taken out, washed with DMF and methanol, and finally dried under a nitrogen stream.

## Supporting Information Available

Materials and reagents, further experimental details, and additional characterization. Figures S1–S23 and Tables S1–S5 (PDF)
