# Supplementary material for: Vapor-Assisted Conversion of Heterobimetallic Titanium–Organic Framework Thin Films
Source: Chem Mater. 2023 Dec 11;35(24):10394–402. doi: 10.1021/acs.chemmater.3c01389 (PMC10853932; doi:10.1021/acs.chemmater.3c01389)
Supplement: Supplementary file 1 — cm3c01389_si_001.pdf [file cm3c01389_si_001.pdf]

SUPPLEMENTARY INFORMATION

Vapor-assisted conversion of Heterobimetallic Titanium-Organic Frameworks thin films

María Romero-Angel,[a] Víctor Rubio-Giménez,[a, b, *] Eloy P. Gómez-Oliveira,[a] Margot F. K. Verstreken,[b] Jorid Smets,[b] Jesús Gándara-Loe,[b] Natalia M. Padial,[a] Rob Ameloot,[b] Sergio Tatay[a, *] and Carlos Martí-Gastaldo[a,*]

[a] Instituto de Ciencia Molecular (ICMol). Universitat de València, Catedrático José Beltrán 2, 46980 Paterna (Spain)

[b] Centre for Membrane Separations, Adsorption, Catalysis and Spectroscopy (cMACS), Katholieke Universiteit Leuven, Celestijnenlaan 200F, 3001 Leuven (Belgium)

S1. MATERIALS AND REAGENTS 1
S2. PHYSICAL CHARACTERIZATION 2
S3. HETEROMETALLIC MUV-101 FILMS THROUGH DIRECT VAPOR ASSISTED CONVERSION (VAC). 3
S4. HETEROMETALLIC MUV-101 FILMS THROUGH METAL-INDUCED DYNAMIC TOPOLOGICAL TRANSFORMATION (MIDTT) 3
S4.1. OPTIMIZATION OF MUV-10 FILMS THROUGH VAPOR ASSISTED CONVERSION 4
S4.1.1. EFFECT OF THE SUBSTRATE 4
S4.1.2. EFFECT OF THE SAM 5
S4.1.3. EFFECT OF THE PRECURSOR CONCENTRATION 7
S4.1.4. EFFECT OF THE MODULATOR 8
S4.1.5. EFFECT OF THE TEMPERATURE AND TIME 10
S4.2. MUV-101 FILMS THROUGH DYNAMIC TOPOLOGYCAL TRANSFORMATION 12
S4.2.1. MUV-10 FILMS 12
S4.2.2. METAL INDUCED DYNAMIC TOPOLOGYCAL TRANSFORMATION 14
S4.3.VERSATILITY OF POST SYNTHETIC MODIFICATION 16
S4.3.1. MUV-102 FILMS THROUGH DYNAMIC TOPOLOGICAL TRANSFORMATION 17
S5. REFERENCES 18

S1. MATERIALS AND REAGENTS

Benzene-1,3,5-tricarboxylic acid (H_3btc, 98%) was purchased from TCI Europe. Anhydrous $CaCl_2$ (≥ 93%), $Co(NO_3)_2 \cdot 6H_2O$ (≥ 98%), $CoCl_2 \cdot 6H_2O$ (99%), $Cu(NO_3)_2 \cdot 3H_2O$ (99%), acetic acid (AcOH, 99%), formic acid (98-100%), benzoic acid (≥ 99.5%) and titanium-(IV) isopropoxide [$Ti(O^{i}Pr)_4$, 97%] were purchased from Sigma-Aldrich. N,N-Dimethylformamide (DMF, ≥ 99.8%), and methanol (≥ 99.9%) were purchased from Scharlab. 4-mercaptopyridine (> 97%) was purchased from TCI Europe. 1-octadecanethiol (> 98%) and 16-mercaptohexadecanoic acid (> 98%) were purchased from Sigma-Aldrich. Ultrapure water with a resistivity higher than 18 MΩ·cm from Milli-Q equipment was used when required. Silicon and silicon oxide (285 nm ±5% wet thermal oxide) substrates were cut to size from single-side polished, p-type B-doped <100>, 6" wafers (NOVA Electronic Materials, Ltd, resistivity = 1−100 Ω cm^{-1}, thickness = 600-700 µm). All other reagents and solvents were used without any previous purification unless specified.

S2. PHYSICAL CHARACTERIZATION

Specular X-ray diffraction (XRD): samples were measured in a PANalytical Empyrean diffractometer (Bragg-Brentano geometry) operating at 40 mA and 45 kVa using copper radiation (Cu Kα = 1.5418 Å) and a PIXcel 1D detector. Profiles were collected in Gonio mode by using a Soller Slit of 0.02° and a divergence slit of ½ at room temperature in the angular range 4° < 2θ < 40° with a step size of 0.013° and a counting time of 36.465 s.

Synchrotron grazing incidence XRD (GIXRD): Measurements were carried out at BL9 beamline of DELTA synchrotron (λ = 1.03 Å; Dortmund, Germany), I07 beamline of Diamond Light Source (λ = 0.99 Å; Didcot, United Kingdom) and the XRD1 beamline of the Elettra Sincrotrone (λ = 1.4 Å; Trieste, Italy) using a stationary MAR or Pilatus 2M detectors, respectively. Thin film samples were placed on holders mounted on multi-axis diffractometers at distances between 350 and 200 mm (all calibrated with LaB_6 reference samples). Multiple pixel images were acquired at various angles of incidence and then processed into diffractograms using FIT2D,[2] DAWN[3] and GIDVis[4] software packages.

Scanning Electron Microscopy (SEM) and Energy Dispersive X-Ray Analysis: Particle morphologies, dimensions, metal-to-metal ratios and elemental mapping were studied with a Hitachi S-4800 SEM at an accelerating voltage of 20 kV. Exclusively for imaging, samples were metalized with a mixture of gold and palladium for 90 s.

Focused ion beam field emission scanning electron microscopy (FIB-FESEM). A Thermo Scientific Scios 2 DualBeam FIB-FESEM was used to cut, image and perform elemental mapping on the isolated crystals deposited on carbon tape. An operating voltage and current of 30 kV and 1 nA were used for cutting and 30 kV and 0.5 nA for polishing the cut area. Then 20 kV and 1.6 nA were used for EDX mapping and 3 kV and 0.2 nA for imaging the inside of the crystals.

Optical Images: images were acquired with a NIKON Eclipse LV-100 optical microscope.

Kr sorption isotherms: The isotherms were measured at 77 K using a Micromeritics 3Flex apparatus. Before the measurement, samples were degassed overnight at 120 ºC under dynamic vacuum (10^{-2} mbar).

S3. HETEROMETALLIC MUV-101 FILMS THROUGH DIRECT VAPOR ASSISTED CONVERSION (VAC).

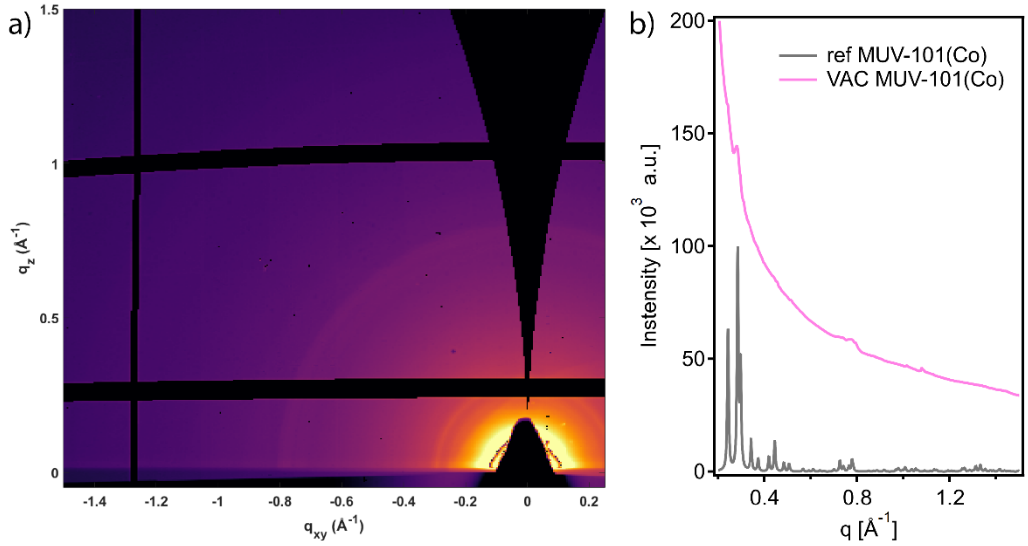

Figure S1. Synchrotron GIXRD of a MUV-101(Co) thin film deposited on a gold-coated substrate directly through VAC. a) Reciprocal space map b) GIXRD diffractogram integrated from the entire reciprocal space map compared to the simulated MUV-101(Co) as a reference.

S4. HETEROMETALLIC MUV-101 FILMS THROUGH METAL-INDUCED DYNAMIC TOPOLOGICAL TRANSFORMATION (MIDTT)

S4.1. OPTIMIZATION OF MUV-10 FILMS THROUGH VAPOR ASSISTED CONVERSION

S4.1.1. EFFECT OF THE SUBSTRATE

Gold-coated silicon substrate without SAM

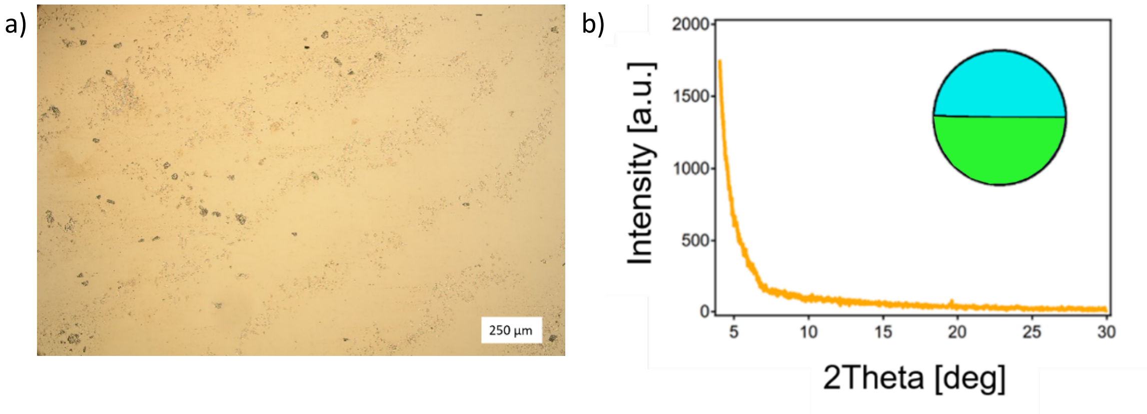

Figure S2. a) Optical image of a MUV-10(Ca) film grown over a gold-coated substrate. b) Specular XRD pattern and film metal composition determined by EDX (Ti in green and Ca in blue).

Silicon substrate without SAM

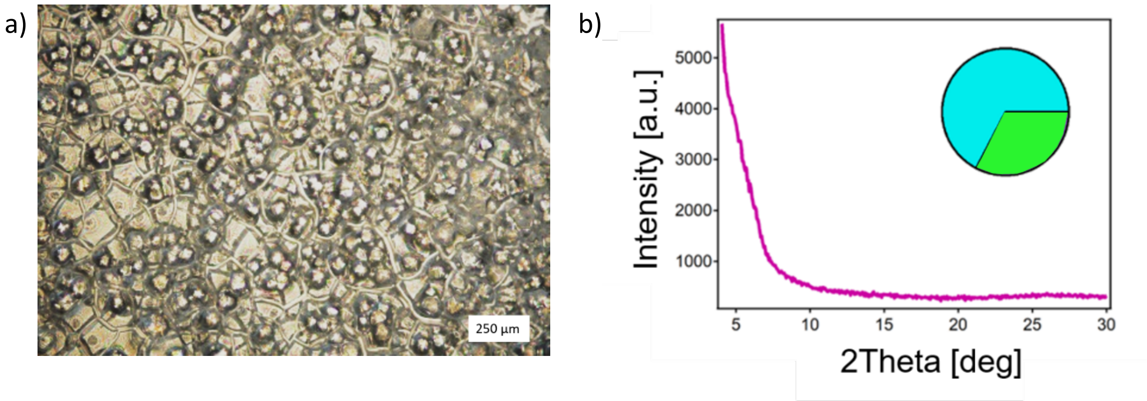

Figure S3. a) Optical image of a MUV-10(Ca) film grown over a Si substrate. b) Specular XRD pattern and film metal composition determined by EDX (Ti in green and Ca in blue).

Silicon oxide substrate without SAM

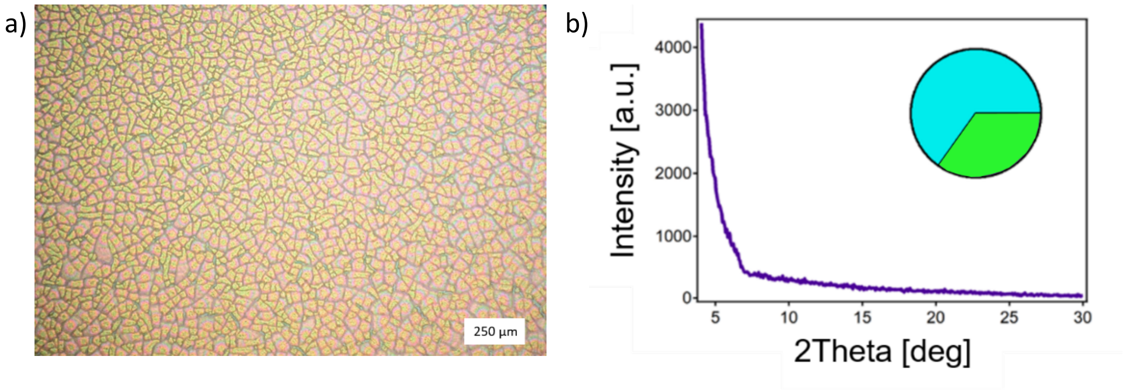

Figure S4. a) Optical image of a MUV-10(Ca) film grown over a SiO_2 substrate. b) Specular XRD pattern and film metal composition determined by EDX (Ti in green and Ca in blue).

S4.1.2. EFFECT OF THE SAM

4-mercaptopyridine SAM

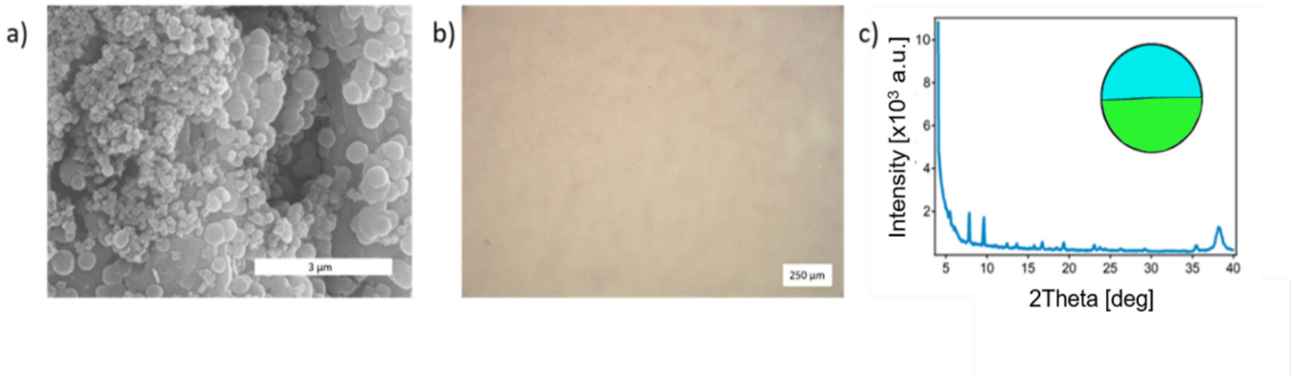

Figure S5. a) SEM image b) Optical image of a MUV-10(Ca) film grown over a gold-coated substrate with a 4-mercaptopyridine SAM. c) Specular XRD pattern and film metal composition determined by EDX (Ti in green and Ca in blue).

1-octadecanethiol SAM

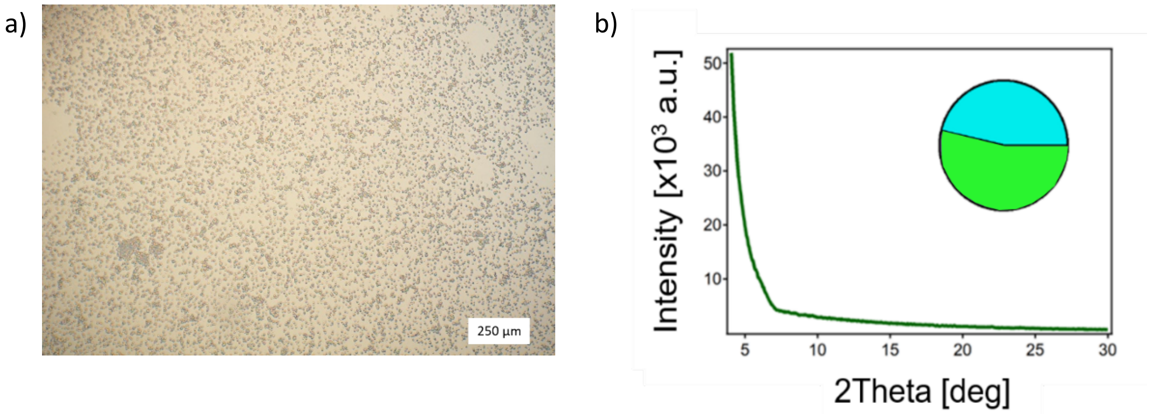

Figure S6. a) Optical image of a MUV-10(Ca) film grown over a gold-coated substrate with a 1-octadecanethiol SAM. b) Specular XRD pattern and film metal composition determined by EDX (Ti in green and Ca in blue).

16-mercaptohexadecanoic acid SAM

a)

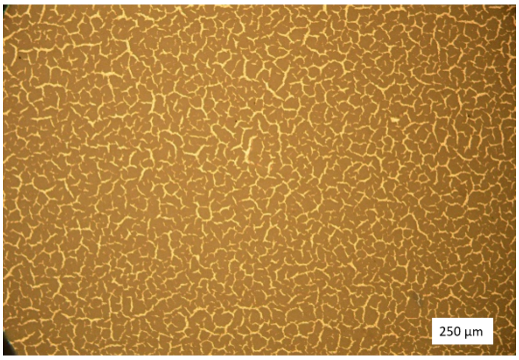

b)

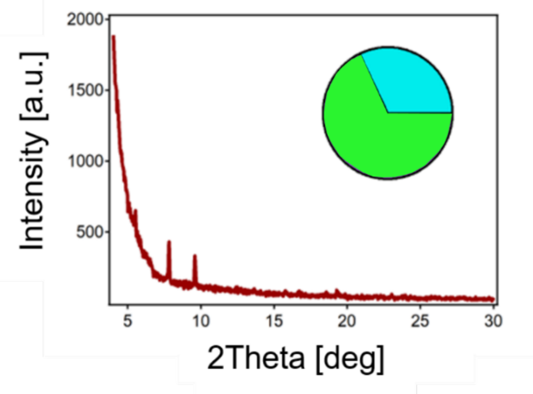

Figure S7. a) Optical image of a MUV-10(Ca) film grown over a gold-coated substrate with a 16-mercaptohexadecanoic acid SAM. b) Specular XRD pattern and film metal composition determined by EDX (Ti in green and Ca in blue).

S4.1.3. EFFECT OF THE PRECURSOR CONCENTRATION

Table S1. Synthetic conditions for the formation of MUV-10(Ca) films.

	Exp	Precursor solution					Droplet volume	Vapor source	
		Ti (IV)	$CaCl_2$	BTC	AcOH	DMF		AcOH	DMF
Variation of precursor and modulator concentration	1	3 µl (0.96 mmol)	1.1 mg (0.96 mmol)	10.5 mg (4.76 mmol)	0 ml (0 eq)	1 ml	50 µl	0.5 ml	2.5 ml
	2	3 µl (0.96 mmol)	1.1 mg (0.96 mmol)	10.5 mg (4.76 mmol)	0.29 ml (500eq)	1ml	50 µl	0.5 ml	2.5 ml
	3	3 µl (0.96 mmol)	1.1 mg (0.96 mmol)	10.5 mg (4.76 mmol)	0.57 ml (1000 eq)	1 ml	50 µl	0.5 ml	2.5 ml
	4	9 µl (3mmol)	3.5 mg (3mmol)	32.4 mg (14.875 mmol)	0 ml (0 eq)	1 ml	50 µl	0.5 ml	2.5 ml
	5	9 µl (3mmol)	3.5 mg (3mmol)	32.4 mg (14.875 mmol)	0.89 ml (500eq)	1 ml	50 µl	0.5 ml	2.5 ml
	**6**	**9 µl (3mmol)**	**3.5 mg (3mmol)**	**32.4 mg (14.875 mmol)**	**1.8 ml (1000 eq)**	**1ml**	**50 µl**	**0.5 ml**	**2.5 ml**
	7	9 µl (3mmol)	3.5 mg (3mmol)	32.4 mg (14.875 mmol)	8 ml (1500 eq)	1 ml	50 µl	0.5 ml	2.5 ml
	8	9 µl (3mmol)	3.5 mg (3mmol)	32.4 mg (14.875 mmol)	10.7 ml (2000eq)	1 ml	50 µl	0.5 ml	2.5 ml
	9	9 µl (3mmol)	3.5 mg (3mmol)	32.4 mg (14.875 mmol)	13.3 ml (2500 eq)	1 ml	50 µl	0.5 ml	2.5 ml
	10	26.5 µl (9 mmol)	10.33 mg (9 mmol)	97 mg (45 mmol)	8 ml (1500 eq)	1ml	50 µl	0.5 ml	2.5 ml
	11	26.5 µl (9 mmol)	10.33 mg (9 mmol)	97 mg (45 mmol)	10.7 ml (2000 eq)	1 ml	50 µl	0.5 ml	2.5 ml
	12	26.5 µl (9 mmol)	10.33 mg (9 mmol)	97 mg (45 mmol)	13.3 ml (2500 eq)	1 ml	50 µl	0.5 ml	2.5 ml
	13	79.5 µl (18 mmol)	31 mg (18 mmol)	291 mg (90 mmol)	16 ml (1500 eq)	1 ml	50 µl	0.5 ml	2.5 ml
	14	79.5 µl (18 mmol)	31 mg (18 mmol)	291 mg (90 mmol)	21.3 ml (2000 eq)	1ml	50 µl	0.5 ml	2.5 ml
	15	79.5 µl (18 mmol)	31 mg (18 mmol)	291 mg (90 mmol)	26.6 ml (2500 eq)	1 ml	50 µl	0.5 ml	2.5 ml
	16	4.5 µl (1.5 mmol)	1.7 mg (1.5 mmol)	15 mg (7.14 mmol)	1.8 ml (1000 eq)	1 ml	50 µl	0.5 ml	2.5 ml
	17	13.3 µl (4.5 mmol)	5 mg (4.5 mmol)	47 mg (22.3 mmol)	1.8 ml (1000 eq)	1 ml	50 µl	0.5 ml	2.5 ml
	18	4.5 µl (1.5 mmol)	1.7 mg(1.5 mmol)	15 mg (7.14 mmol)	8 ml (1500 eq)	1ml	50 µl	0.5 ml	2.5 ml
	19	13.3 µl (4.5 mmol)	5 mg (4.5 mmol)	47 mg (22.3 mmol)	8 ml (1500 eq)	1 ml	50 µl	0.5 ml	2.5 ml
	20	4.5 µl (1.5 mmol)	1.7 mg (1.5 mmol)	15 mg (7.14 mmol)	0.89 ml (500 eq)	1 ml	50 µl	0.5 ml	2.5 ml
	21	13.3 µl (4.5 mmol)	5 mg (4.5 mmol)	47 mg (22.3 mmol)	0.89 ml (500 eq)	1 ml	50 µl	0.5 ml	2.5 ml

S4.1.4. EFFECT OF THE MODULATOR

Gold-coated substrate with 4-mercaptopyridine SAM, Modulator benzoic acid

a) b)

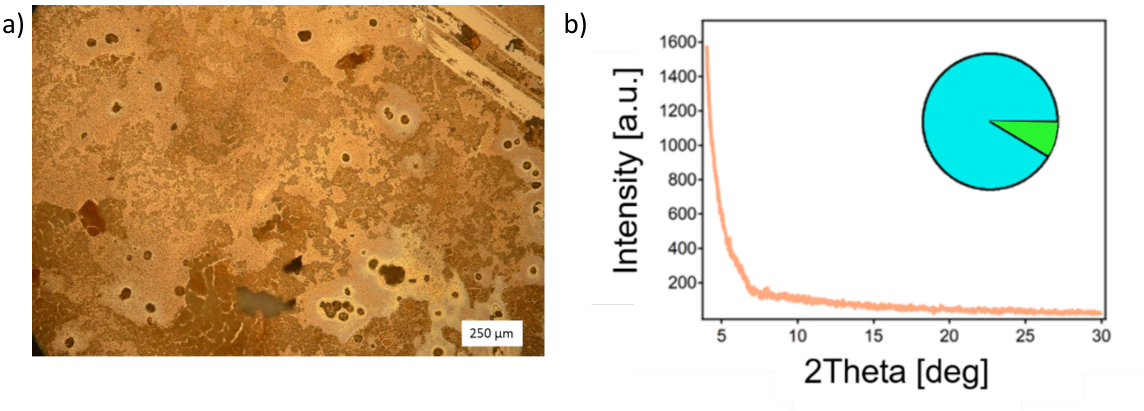

Figure S8. a) Optical image of a MUV-10(Ca) film grown over a gold-coated substrate with a 4-mercaptopyridine SAM and benzoic acid as modulator. b) Specular XRD pattern and film metal composition determined by EDX (Ti in green and Ca in blue).

Gold-coated substrate with 4-mercaptopyridine SAM, Modulator formic acid

a) b)

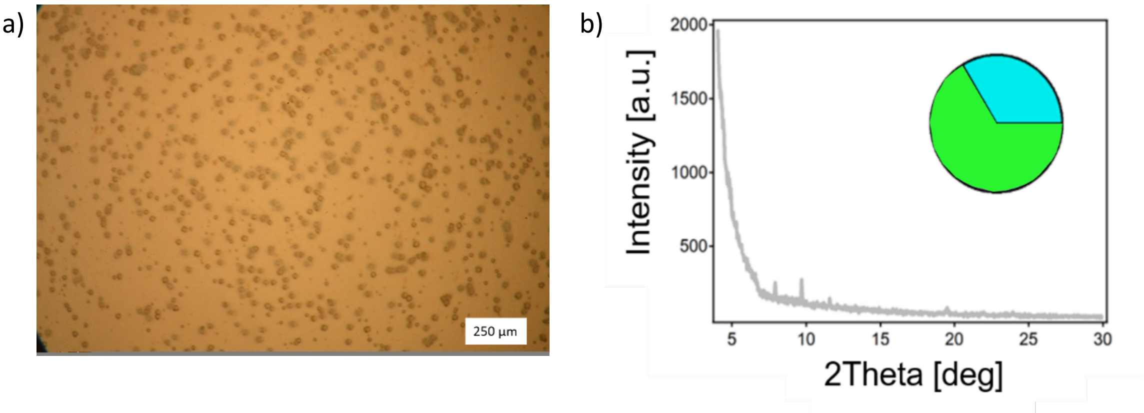

Figure S9. a) Optical image of a MUV-10 (Ca) film grown over a gold-coated substrate with a 4-mercaptopyridine SAM and formic acid as modulator. b) Specular XRD pattern and film metal composition determined by EDX (Ti in green and Ca in blue).

Gold-coated substrate with 4-mercaptopyridine SAM, Modulator acetic acid

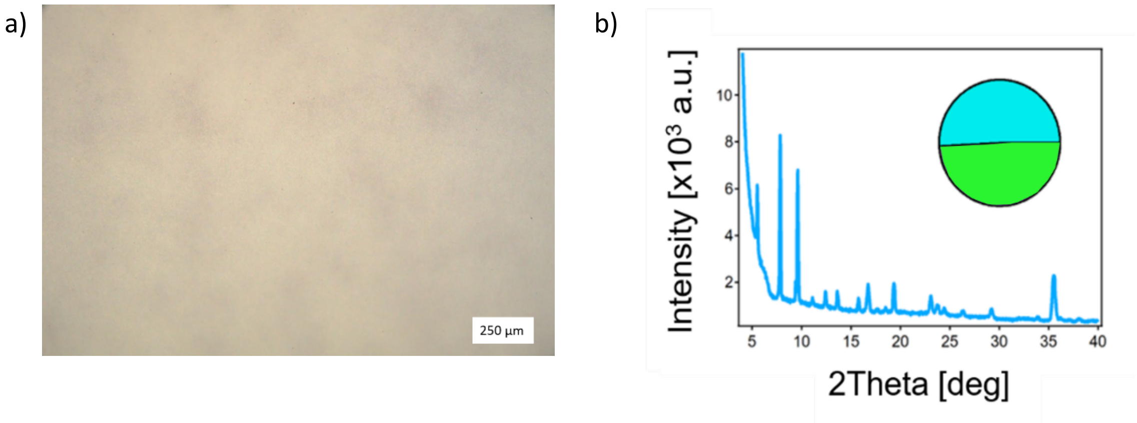

Figure S10. a) Optical image of a MUV-10(Ca) film grown over a gold-coated substrate with a 4-mercaptopyridine SAM and AcOH as modulator. b) Specular XRD pattern and film metal composition determined by EDX (Ti in green and Ca in blue).

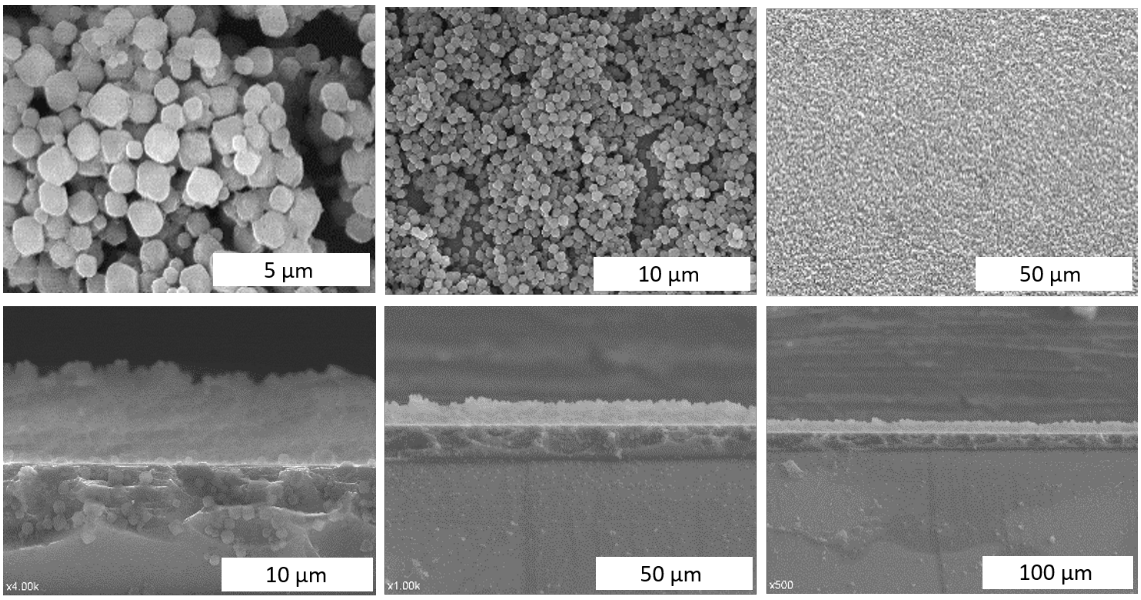

Figure S11. SEM imaging of the top (top) and cross-section (bottom) of a MUV-10(Ca) film grown over a gold-coated substrate with a 4-mercaptopyridine SAM and AcOH as modulator.

S4.1.5. EFFECT OF THE TEMPERATURE AND TIME

The reactions were carried out by following the original published conditions.[1]

Modulator benzoic acid, 120ºC, 48h

a) 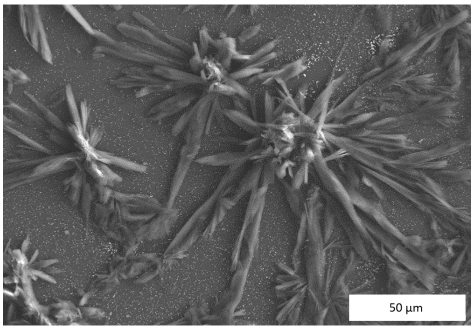

b) 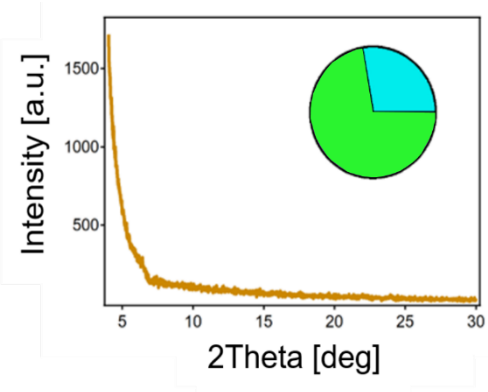

Figure S12. a) SEM image of a MUV-10 (Ca) film grown over a gold-coated substrate with a 4-mercaptopyridine SAM, benzoic acid as modulator and 120ºC for 48h. b) Specular XRD pattern and film metal composition determined by EDX (Ti in green and Ca in blue).

Modulator acetic acid, 120ºC, 48h

a) 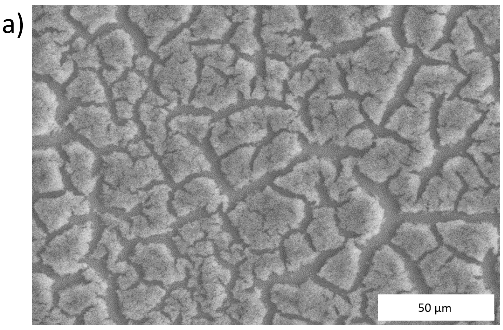

b) 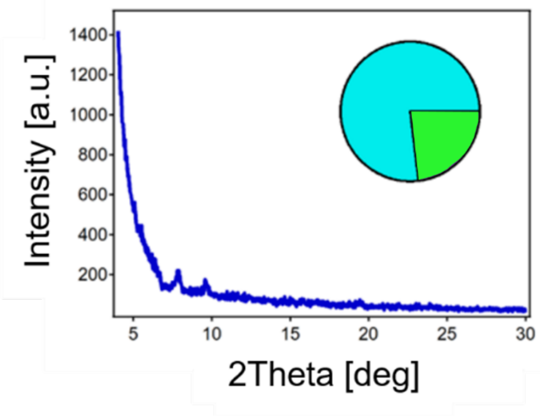

Figure S13. a) SEM image of a MUV-10 (Ca) film grown over a gold-coated substrate with a 4-mercaptopyridine SAM, AcOH as modulator and 120ºC for 48h. b) Specular XRD pattern and film metal composition determined by EDX (Ti in green and Ca in blue).

Modulator formic acid, 120ºC, 48h

a) b)

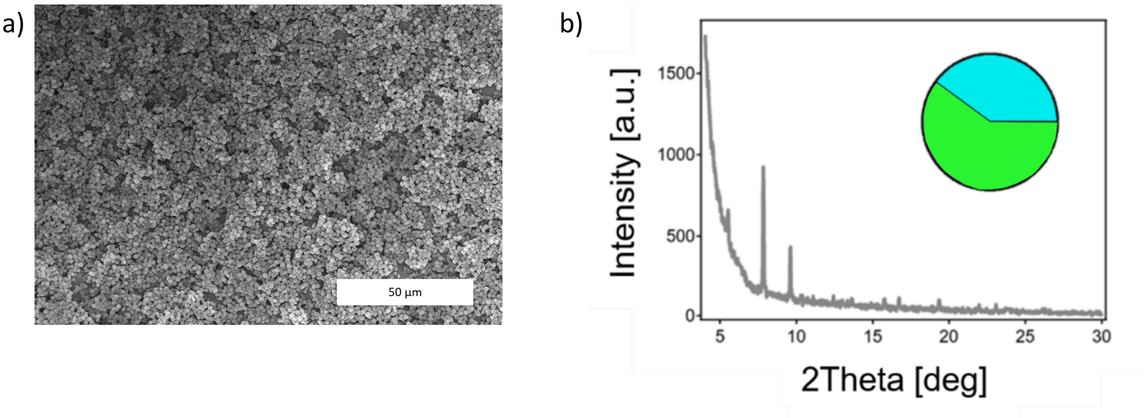

Figure S14. a) SEM image of a MUV-10 (Ca) film grown over a gold-coated substrate with a 4-mercaptopyridine SAM, formic acid as modulator and 120ºC for 48h. b) Specular XRD pattern and film metal composition determined by EDX (Ti in green and Ca in blue).

S4.2. MUV-101 FILMS THROUGH DYNAMIC TOPOLOGYCAL TRANSFORMATION

S4.2.1. MUV-10 FILMS

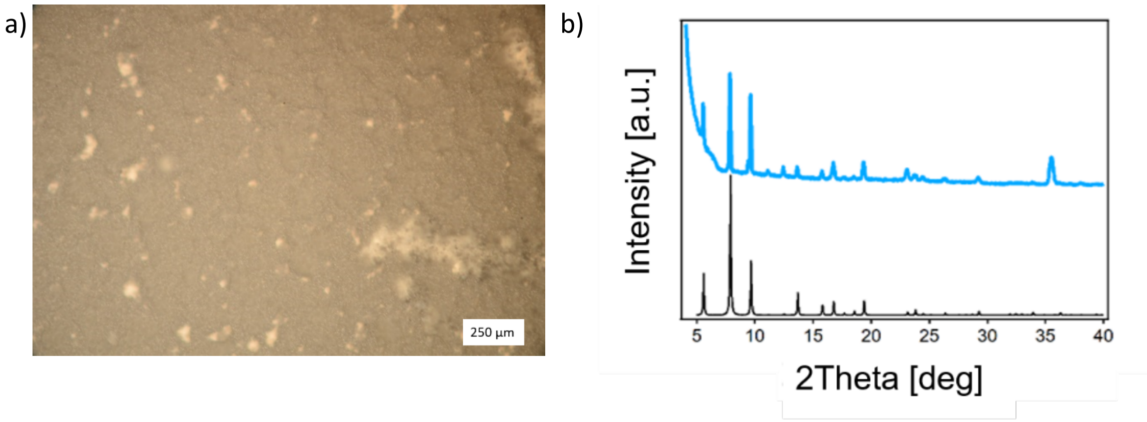

Figure S15. a) Optical image of the optimized MUV-10(Ca) film. b) Specular XRD pattern of the film comparison with the simulated MUV-10 (black).

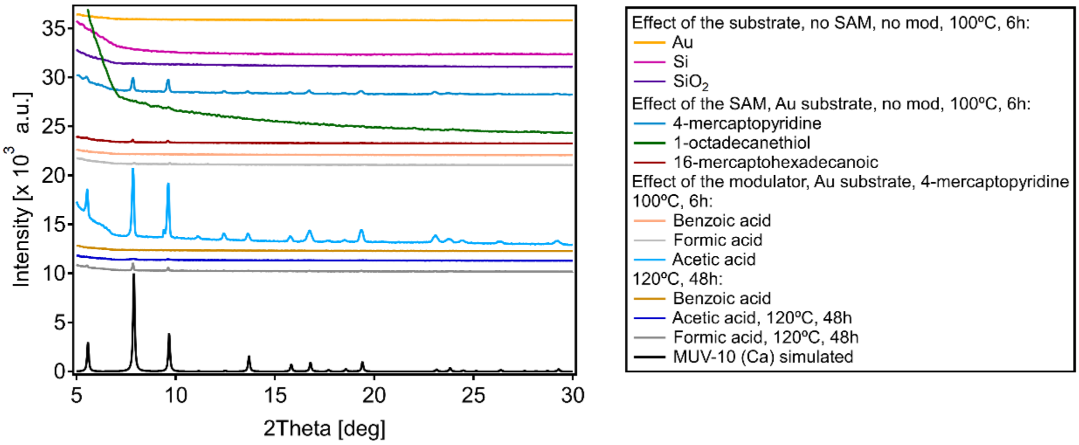

Figure S16. Comparison of the specular XRD patterns of all the above MUV-10(Ca) VAC conditions and the simulated MUV-10 (black).

Scanning Electron Microscopy and Energy-Dispersive X-Ray Analysis

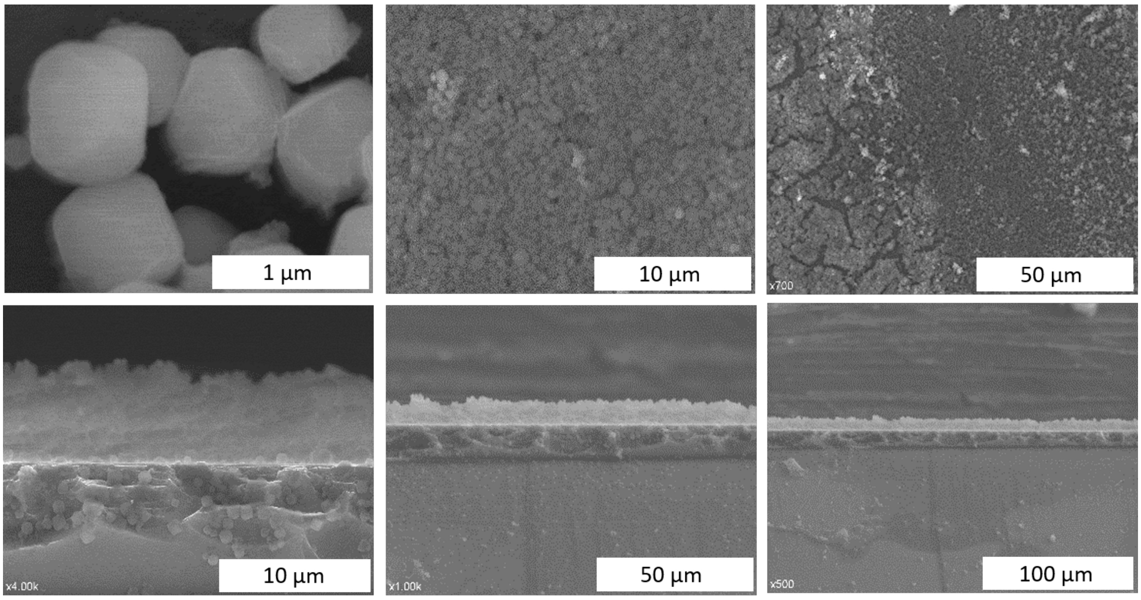

Figure S17. SEM imaging of the top (top) and cross-section (bottom) of the optimized VAC MUV-10(Ca) film.

S4.2.2. METAL INDUCED DYNAMIC TOPOLOGYCAL TRANSFORMATION

Scanning Electron Microscopy and Energy-Dispersive X-Ray Analysis

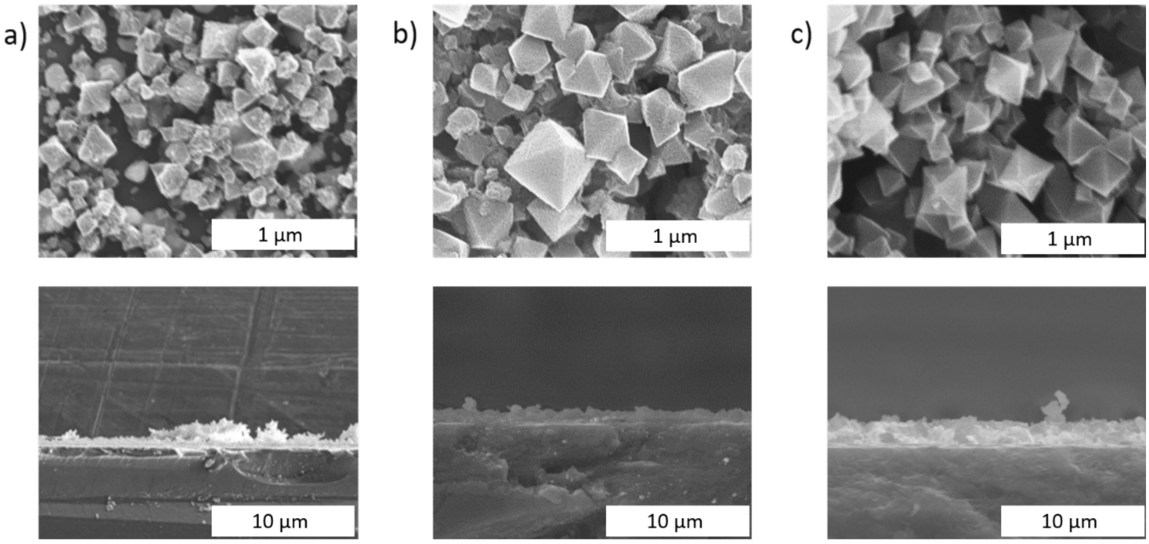

Figure S19. SEM imaging of the top (top) and cross-section (bottom) of MUV-101(Co) films at a) the beginning (1 h), b) in the middle of the reaction (5 days) and c) at the end of the transformation (15 days).

Table S2. EDX of MUV-101(Co) films fabricated by MIDTT at different times.

Sample	Ca [at. %]	Ti [at. %]	Co [at. %]
MUV-101(Co)-1 hour	50.60	48.56	0.84
MUV-101(Co)-5 hour	47.74	50.15	2.11
MUV-101(Co)-12 hour	40.96	50.98	8.06
MUV-101(Co)-1 day	28.41	53.32	18.27
MUV-101(Co)-2 days	22.36	54.89	22.75
MUV-101(Co)-5 days	16.67	55.98	27.35
MUV-101(Co)-10 days	0.20	54.57	45.23
MUV-101(Co)-15 days	0.32	50.85	48.83
MUV-101(Co)-20 days	0.03	46.11	53.86
MUV-101(Co)-30 days	0.01	45.82	54.17

Table S3. First order rate constants for the formation of MUV-101(Co) films via MIDTT as determined from EDX atomic percentages data.

Metal	Rate constants (days^{-1})
**Ca**	0.4 ± 0.1
**Ti**	0.20 ± 0.04
**Co**	0.16 ± 0.05

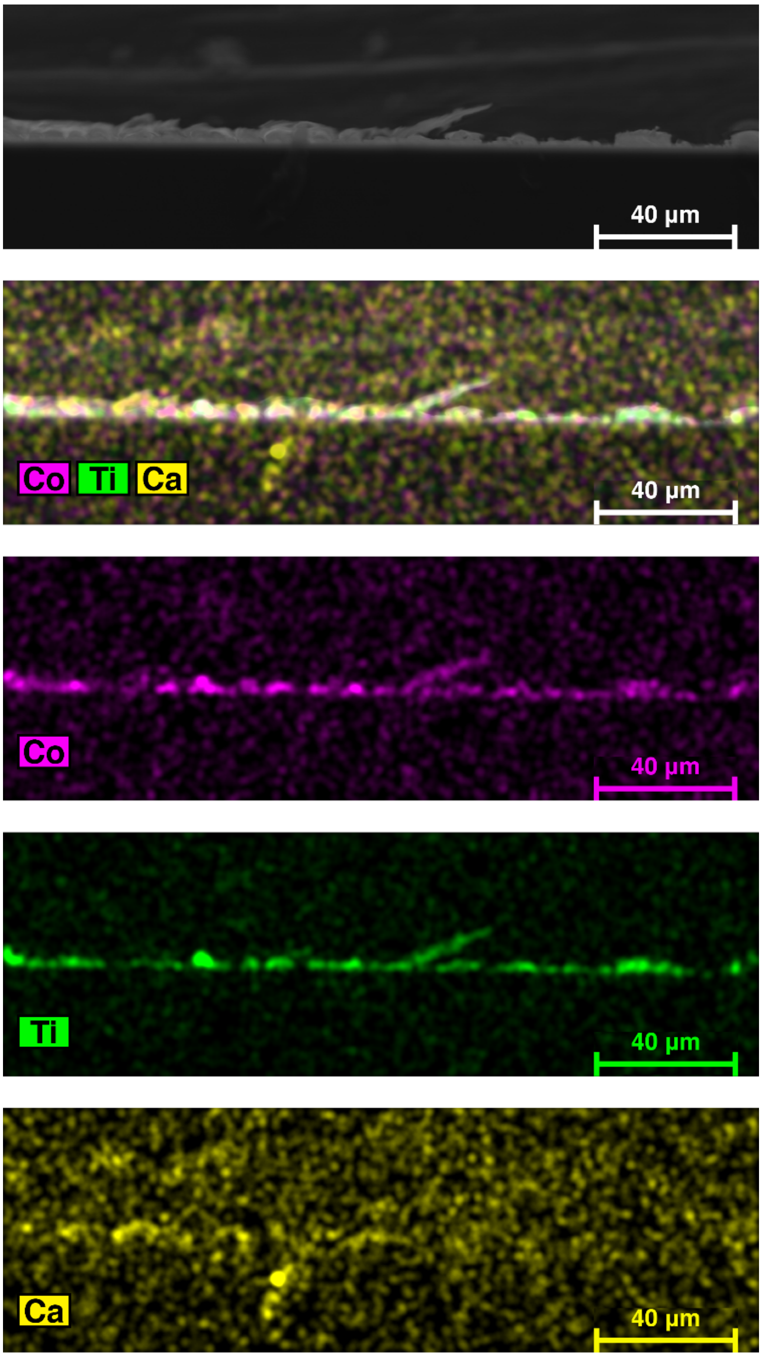

Figure S20. SEM-EDX imaging and mapping of the cross-section of a MUV-101(Co) film at the end of the MIDTT (15 days). Color code: Co, pink; Ti, green; Ca, yellow.

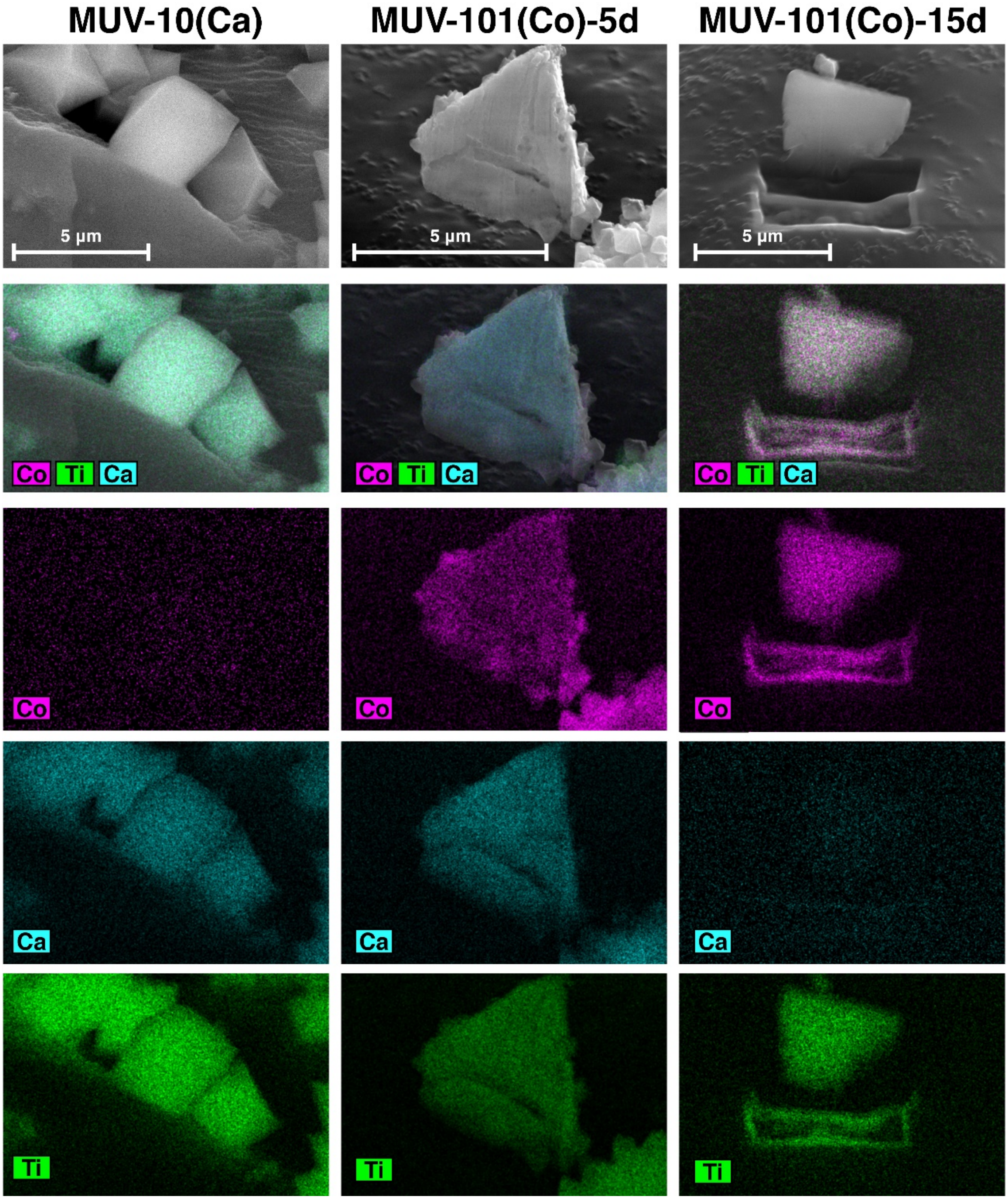

Figure S21. FIB-FESEM imaging and EDX mapping of a MUV-10(Ca) crystal transformation into MUV-101(Co), including the initial (0 days), intermediate (5 days) and final (15 days) stages of MIDTT. Color code: Co, pink; Ti, green; Ca, yellow.

S4.3.

VERSATILITY OF POST SYNTHETIC MODIFICATION

S4.3.1. MUV-102 FILMS THROUGH DYNAMIC TOPOLOGICAL TRANSFORMATION

Scanning Electron Microscopy and Energy-Dispersive X-Ray Analysis

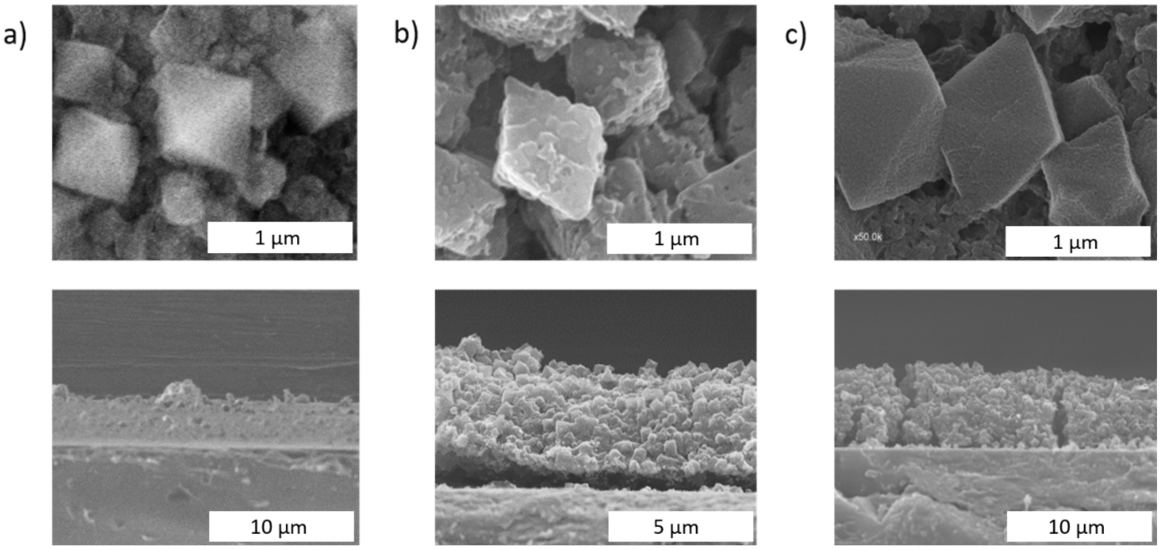

Figure S22. SEM imaging of the top (top) and cross-section (bottom) of MUV-102(Cu) films from the top and in profile at a) the beginning (1 h), b) in the middle of the reaction (1 day) and c) at the end of the transformation (3 days).

Table S4. EDX of MUV-102(Cu) films fabricated by MIDTT at different times.

Sample	Ca [at. %]	Ti [at. %]	Cu [at. %]
MUV-102(Cu)-1 hour	35.62	50.85	13.52
MUV-102(Cu)-5 hour	29.46	49.26	21.28
MUV-102(Cu)-12 hour	23.69	48.12	28.19
MUV-102(Cu)-1 day	11.11	47.31	41.58
MUV-102(Cu)-2 days	6.89	35.59	57.52
MUV-102(Cu)-3 days	3.95	26.30	69.75
MUV-102(Cu)-5 days	1.79	27.75	70.46
MUV-102(Cu)-10 days	0.05	26.84	73.11
MUV-102(Cu)-15 days	0.04	26.66	73.30

Table S5. First order rate constants for the formation of MUV-102(Cu) films via MIDTT as determined from EDX atomic percentages data.

Metal	Rate constants ($days^{-1}$)
**Ca**	0.5 ± 0.1
**Ti**	0.2 ± 0.1
**Cu**	0.4 ± 0.1

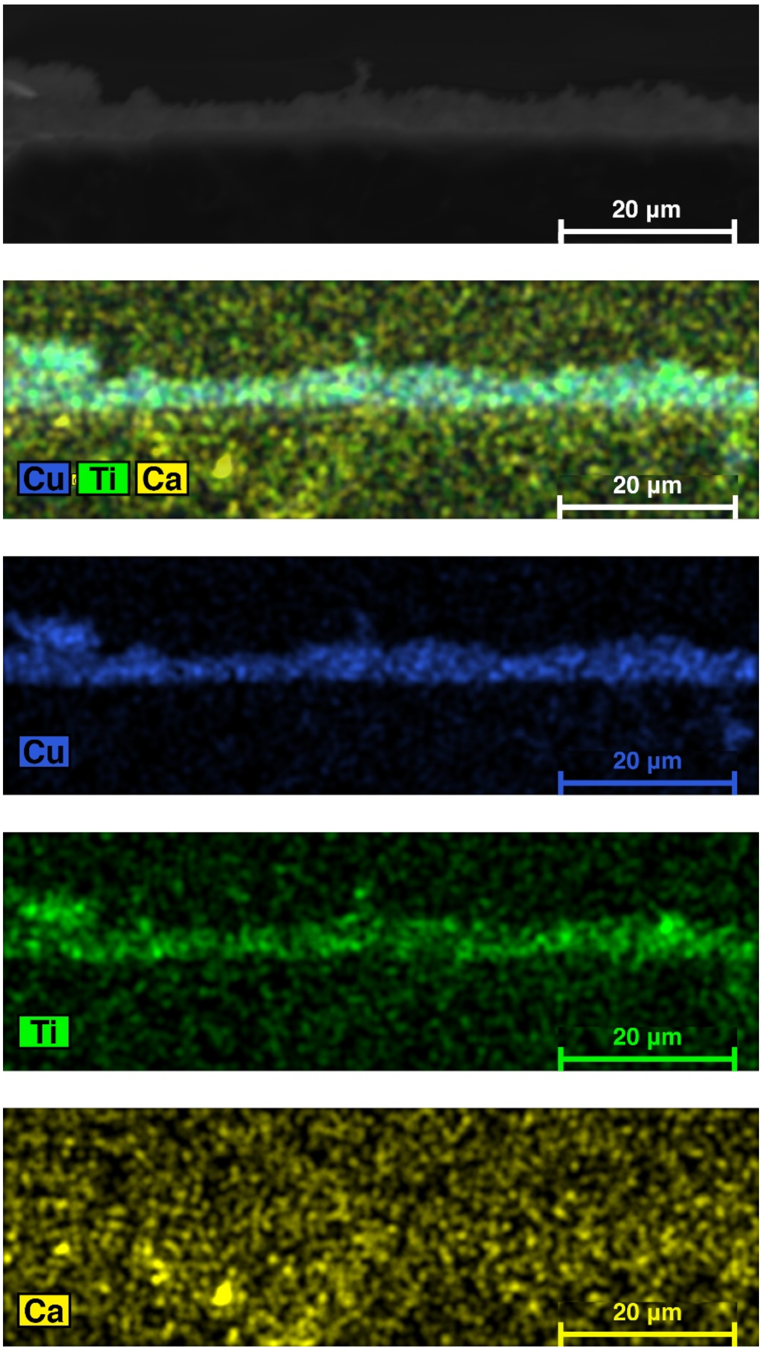

Figure S23. SEM-EDX imaging and mapping of the cross-section of a MUV-102(Cu) film at the end of the MIDTT (5 days). Color code: Co, pink; Ti, green; Ca, yellow.

S5. REFERENCES

1 J. Castells-Gil, N. M. Padial, N. Almora-Barrios, R. Gil-San-Millán, M. Romero-Ángel, V. Torres, I. da Silva, B. C. J. Vieira, J. C. Waerenborgh, J. Jagiello, J. A. R. Navarro, S. Tatay and C. Martí-Gastaldo. *Chem*. **2020**, *6*, 3118-3131

2 A. P. Hammersley, S. O. Svensson, M. Hanfland, A. N. Fitch and D. Häusermann. *High Pressure Research.* **2006**, *14*, 235–248.

3 J. Filik, A. W. Ashton, P. C. Y. Chang, P. A. Chater, S. J. Day, M. Drakopoulos, M. W. Gerring, M. L. Hart, O. V. Magdysyuk, S. Michalik, A. Smith, C. C. Tang, N. J. Terrill, M. T. Wharmby and H. Wilhelm. *J. Appl. Cryst.* **2017**, *50*, 959–966.

4 B. Schrode, S. Pachmajer, M. Dohr, C. Röthel, J. Domke, T. Fritz, R. Resel and O. Werzer. *J. Appl. Cryst.* **2019**, *52*, 683–689.
